# Commercial volumetric MRI reporting tools in multiple sclerosis: a systematic review of the evidence

**DOI:** 10.1007/s00234-022-03074-w

**Published:** 2022-11-04

**Authors:** Zoe Mendelsohn, Hugh G. Pemberton, James Gray, Olivia Goodkin, Ferran Prados Carrasco, Michael Scheel, Jawed Nawabi, Frederik Barkhof

**Affiliations:** 1 Neuroradiological Academic Unit, UCL Queen Square Institute of Neurology, University College London, London, UK; 2 Department of Medical Physics and Bioengineering, Centre for Medical Image Computing (CMIC), University College London, London, UK; 3 Department of Neuroinflammation, Queen Square Multiple Sclerosis Centre, UCL Institute of Neurology, University College London, London, UK; 4 Department of Neuroradiology, Charité School of Medicine and University Hospital Berlin, Berlin, Germany; 5 Department of Radiology, Charité School of Medicine and University Hospital Berlin, Berlin, Germany; 6 GE Healthcare, Amersham, UK; 7 Stepping Hill Hospital, NHS Foundation Trust, Stockport, UK; 8 E-Health Centre, Universitat Oberta de Catalunya, Barcelona, Spain; 9 Berlin Institute of Health at Charité – Universitätsmedizin Berlin, BIH Biomedical Innovation Academy, BIH Charité Digital Clinician Scientist Program, Berlin, Germany; 10 Radiology & Nuclear Medicine, Amsterdam University Medical Centers, Vrije Universiteit, Amsterdam, The Netherlands

**Keywords:** Systematic review, Multiple sclerosis, MRI, Quantitative volumetric reporting tools, Validation

## Abstract

**Purpose:**

MRI is integral to the diagnosis of multiple sclerosis (MS) and is important for clinical prognostication. Quantitative volumetric reporting tools (QReports) can improve the accuracy and objectivity of MRI-based assessments. Several QReports are commercially available; however, validation can be difficult to establish and does not currently follow a common pathway. To aid evidence-based clinical decision-making, we performed a systematic review of commercial QReports for use in MS including technical details and published reports of validation and in-use evaluation.

**Methods:**

We categorized studies into three types of testing: technical validation, for example, comparison to manual segmentation, clinical validation by clinicians or interpretation of results alongside clinician-rated variables, and in-use evaluation, such as health economic assessment.

**Results:**

We identified 10 companies, which provide MS lesion and brain segmentation and volume quantification, and 38 relevant publications. Tools received regulatory approval between 2006 and 2020, contextualize results to normative reference populations, ranging from 620 to 8000 subjects, and require T1- and T2-FLAIR-weighted input sequences for longitudinal assessment of whole-brain volume and lesions. In MS, six QReports provided evidence of technical validation, four companies have conducted clinical validation by correlating results with clinical variables, only one has tested their QReport by clinician end-users, and one has performed a simulated in-use socioeconomic evaluation.

**Conclusion:**

We conclude that there is limited evidence in the literature regarding clinical validation and in-use evaluation of commercial MS QReports with a particular lack of clinician end-user testing. Our systematic review provides clinicians and institutions with the available evidence when considering adopting a quantitative reporting tool for MS.

## Introduction

Multiple sclerosis (MS) is a chronic inflammatory and neurodegenerative disease of the central nervous system characterized by demyelinating lesions and atrophy [1, 2]. Brain atrophy is accelerated in MS compared to the healthy population [3]. Both lesion evolution and brain volume loss over time correlate with clinical disability [3, 4].

Structural MRI is routinely used in the diagnostic workup of MS and to assess and monitor demyelinating lesions [5]. MRI-based measurement of brain atrophy is becoming increasingly recognized as an important clinical prognostication tool [3, 6, 7]. Brain and lesion volumes measured using image segmentation have become established biomarkers for determining treatment efficacy in research studies and clinical trials [8–11]. Previous research has shown that brain atrophy [12] and lesion volumes [13] significantly predict long-term disability in all MS phenotypes, especially when used in combination [4, 9]. Manual segmentation of the brain and lesions is time-consuming and can also be prone to imprecision and error [14–16]. The development and use of automated and semi-automated brain and lesion segmentation methods, such as in quantitative volumetric reporting tools (QReports), has increased in recent years [11, 17]. These tools aim to improve the objectivity of image interpretation by increasing the sensitivity of MRI analysis [18, 19], the accuracy[20–25] and reproducibility of results [22, 26], and potentially decreasing reporting time [18]. QReports can facilitate cross-sectional diagnosis [20, 26–30], longitudinal assessment [20, 22, 23, 31], and therapy response monitoring [32] via user-friendly graphical displays. QReports may also offer automatic contextualization of an individual patient’s volumetric results against a relevant reference population [33], which could assist clinicians in disease course prognostication and deciding on therapeutic strategies. Various QReports for MS have been developed for use in the clinical setting, and many of these tools are commercially available having received regulatory approval.

Currently, the application of QReports in the clinic is limited [11, 12]. Clinical institutions may not have adequate resources to assess how tools have been tested and validated, despite commercialization for medical use. To encourage evidence-based use and to aid clinicians in deciding how and whether to adopt these tools, the validity of results and the impact on clinical management should be established. Technical and clinical validation and evaluation of quantitative reporting tools do not currently follow standardized methods. The quantitative neuroradiology initiative (QNI) addresses this issue and provides a six-step translational pathway for quantitative reporting tools [34]. The QNI model distinguishes three types of testing: technical validation of tool performance, for example, comparison to manual segmentation or other segmentation techniques; clinical validation by clinicians or by interpretation of results alongside clinician-rated variables; and finally in-use evaluation, such as health economic assessment [34].

Our previous work demonstrated a lack of technical and notably clinical validation of commercial QReports in dementia [35]. In the current paper, we replicated this methodology and performed a systematic review of the literature aiming to validate or evaluate commercial QReports for use in MS. We (1) presented the range of tools, including details of their technical features and characteristics and (2) provided a descriptive synthesis of the evidence published regarding their validation. We assessed the literature according to the QNI framework, categorizing studies into technical and clinical validation and in-use evaluation. The aim is to increase transparency and help clinicians to make informed decisions about whether to adopt commercial QReports into clinical routine for the assessment of patients with MS and provide an overview of the features of each commercially available tool.

## Methods

This review was conducted according to the Preferred Reporting Items for Systematic Reviews and Meta-Analyses (PRISMA) guidelines [36–38] and is registered with the Prospective Register of Systematic Reviews (PROSPERO) database under number CRD42021286139.

### Vendor and product search

#### Product inclusion and exclusion criteria

The inclusion criteria for QReports are as follows: (1) FDA or CE clearance; (2) target disorder MS or a population with suspected MS (specified on the company website or in the literature); (3) uses structural-MRI-based input (4) to generate brain and lesion volumetric results; (5) incorporates normative reference data for single-subject comparison; (6) presents results in a structured report format.

#### Search methodology: FDA-cleared product identification

1. Keyword screening

The FDA medical device databases were used (last access: 28 January 2022) to find FDA-cleared automated quantitative MRI reporting tools in MS (https://www.fda.gov/medical-devices/device-advice-comprehensive-regulatory-assistance/medical-device-databases). A total of 83,556 premarket 510(k) FDA notification clearances dating from 1996 to present were downloaded in a text file from https://www.fda.gov/medical- devices/510k-clearances/downloadable-510k-files. The text file was searched using the keywords listed below and 821 “medical devices” were identified for further review. Terms with an * use “wild-cards,” covering relevant suffixes of each word stem, for example, “Radiolog*” covers “Radiology,” “Radiologist,” and “Radiological”:

**Table:** 

• Neuro*	• Cortex	• Structur*
• Brain	• Dementia	• Segment*
• Quant*	• Volume	• Automat*
• MRI	• Multiple	• Spinal
• Hippocamp*	• Sclerosis	• Cord
• Radiolog*	• Lesion	• MS
• Atroph*	• Lobar	• Demyelinat*
• Cortical	• Lobe

2. Eligibility screening

**Fig. 1:**
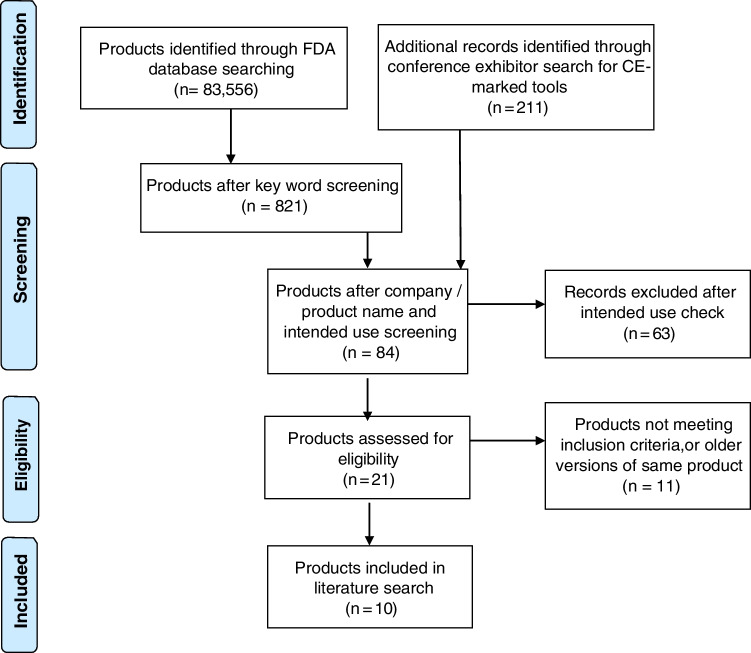
PRISMA flow diagram outlining a systematic search for CE- and FDA-approved QReports. The FDA medical device databases and lists of all companies that exhibited at relevant medical imaging conferences (ISMRM, ESMRMB, RSNA, ECR, ESR AIX, ASNR, SIIM, and ESNR) were searched and the website https://grand-challenge.org/aiforradiology/ was used to cross-check results

Manual checks were performed to verify the company name, product name, approval date, and description on the FDA database. Tools considered hardware were excluded at this stage. The websites of all remaining companies were searched to further investigate the intended use of their products. Seven companies that had not specified MS as the target disorder were excluded from further review. Two quantitative reporting tools that were acquisition dependent were also excluded at this stage. After manual checks and searching company websites, four companies were identified as meeting our inclusion criteria (see Fig. 1 for PRISMA flowchart outlining search for companies).

#### Search methodology: CE-marked product identification

As there was no freely available, searchable database of CE-marked products, the websites of relevant medical imaging conferences (ISMRM, ESMRMB, RSNA, ECR, ESR AIX, ASNR, SIIM, and ESNR) were searched to identify companies that exhibited their products in 2019–2021. The websites of identified companies were searched in detail to find CE-marked quantitative reporting tools. Sixty-three tools were excluded after screening the company and product name and intended use. Results were cross-checked using the following website: https://grand-challenge.org/aiforradiology/. Two companies that had not specified MS as the target disorder were excluded from further review. Finally, six companies with CE-marked tools were identified that fit our inclusion criteria.

Each company was directly contacted to verify the product name, date of approval, description, and intended use of the product. The companies were informed of their inclusion in the review and given the opportunity to add to and correct information gathered from company websites and the literature. Excluded companies are summarized in the “Results” section.

### Company and product features

The following technical details of the 10 tools included in the review were extracted from company websites, publications identified in the literature search, and by direct vendor contact:

- FDA/CE approval
- Date of approval
- Target disorder
- Input sequences
- Brain and lesion segmentation and volumetry method
- Lesion filling
- Brain atrophy data
- Cross-sectional or longitudinal analysis available
- Details of normative reference populations
- Provision of segmentation overlays
- Strategies to account for inter-scanner variability
- Image quality control method(s)
- Report deployment procedure

### Literature search on technical and clinical validation of identified products

A literature search was conducted independently by two authors according to PRISMA guidelines [36–38]. The results were checked and verified by a third author; any inclusion or exclusion discrepancies were settled by consensus. The 10 company names and their associated product names were used as search terms. Both company and product names were used to ensure the identification of studies published before product branding. Searches were simultaneously conducted in PubMed, Ovid Medline “All fields” and Scopus (latest search: 29 March 2022). Company and product names comprising several words are bracketed to indicate single search terms. Companies were directly contacted to verify company and product names. The search terms were as follows:

1. AIRAmed *OR* (AIRAscore)
2. Combinostics *OR* (cNeuro cMRI)
3. (CorTechs Labs) *OR* (NeuroQuant MS) *OR* LesionQuant
4. Icometrix *OR* MSmetrix *OR* (icobrain ms)
5. (Jung diagnostics) *OR* Biometrica
6. mediaire *OR* mdbrain
7. Pixyl *OR* Pixyl.Neuro.MS *OR* Pixyl.Neuro.BV
8. Quibim *OR* (Quibim Precision)
9. Qubiotech *OR* (Neurocloud VOL)
10. Qynapse *OR* QyScore

References in identified publications, papers listed under “similar articles” in PubMed, and all publications listed on company websites were also searched to identify additional relevant validation studies. Companies were given the opportunity to provide further relevant studies.

### Study inclusion criteria

The study inclusion criteria used in this review are based on the QNI framework for the translation of quantitative reporting tools into the clinic. Studies were included in the review on the basis that they met the following inclusion criteria: (1) published in English as original research in academic peer-reviewed journals or conference proceedings (conference abstracts and posters excluded), (2) which involve automated lesion, or brain *and* lesion, segmentation and volumetry computed from structural MR images (3) in an MS population and/or healthy controls, and (4) fit either:

#### Technical validation

Papers validating the technical performance of lesion or brain *and* lesion segmentation methods. For example, test-retest studies or comparison to manual segmentation and/or other state-of-the-art brain volumetry tools, such as FreeSurfer [39], SPM (www.fil.ion.ucl.ac.uk/spm), SIENA(X) [40], and lesion segmentation tools, for example, LST [41, 42], nicMSlesions [43], and samseg [44], and testing for robustness to different input data. Papers focusing only on brain segmentation were not included unless conducted in an MS population.

#### Clinical validation

1. Testing of the tool by clinicians on an MS population focusing on one of more of the following: (a) determining diagnostic accuracy, confidence, and differential diagnoses vs. “ground truth” clinician-rated diagnoses, i.e., using receiver operating characteristics; (b) assessing the tool’s effect on clinical management (usability, prognostic value); (c) inter-rater reliability metrics or percent agreement.
2. Clinical trials in MS using the tool’s results as an outcome measurement.
3. Interpretation of results alongside clinician-rated variables, such as the Expanded Disability Status Scale (EDSS) and Symbol Digit Modalities Test (SDMT), as measures of physical and cognitive disability in MS, respectively.

#### In-use evaluation

Studies assessing any of (1) benefit to patients, (2) the effect on reporting time in the context of normal clinical routine, (3) clinical and population perception, or (4) socioeconomic impact of using QReports in the clinic.

### Data extraction

Two raters independently categorized all studies that met our inclusion criteria into technical validation, clinical validation, or in-use evaluation. A third researcher checked the results, and a consensus was reached on any discrepancies.

## Results

### Company and product search

Following the systematic search outlined above, 10 companies were identified that produce tools meeting our inclusion criteria; see Fig. 1 for a research flow diagram summarizing the search for relevant products.

#### Excluded tools

According to PRISMA guidelines, inclusion criteria were decided on in advance (see “Methods” section). The results of the eligibility screening are presented below.

MS brain and lesion segmentation and volumetry tools were excluded if they were not FDA or CE approved, such as SegPlus by Neurophet (https://www.neurophet.com), which has conducted technical validation [45], and TensorMedical (https://www.tensormedical.ai) that developed and uses the nicMSlesions software and has evidence of technical validation in MS [43]. Research tools that did not have FDA or CE approval, such as FreeSurfer [39], SPM (www.fil.ion.ucl.ac.uk/spm) or SIENA(X) [40] and LST [41, 42], samseg [44], or BaMoS [46], were excluded.

QReports that, according to the literature and the company websites, did not conduct brain and MS lesion segmentation, including Childmetrix by Icometrix (a pediatric non-MS-related QReport) (http://icometrix.com), Quantib ND by Quantib (https://www.quantib.com), neuroreader by Brainreader (https://brainreader.net), THINQ by Corticometrics (https://www.corticometrics.com), tools by JLK Inc (https://www.jlkgroup.com), and Corinsights MRI by ADM diagnostics (https://admdx.com), VUNO Med-DeepBrain by Vuno (https://www.vuno.co), AI-Rad Companion Brain by Siemens Health (https://www.siemens-healthineers.com), AQUA by Neurophet (https://www.neurophet.com), and DIADEM by Brainminer (https://www.brainminer.co.uk), were excluded. Our research group has conducted a systematic review including several of these tools for other indications [35].

Acquisition-dependent quantitative neuroimaging tools were also excluded, including SyMRI Neuro by SyntheticMR (https://syntheticmr.com/) and STAGE by SpinTechMRI (https://spintechmri.com), which include dedicated quantitative MRI-based reporting tools that can be used in the setting of MS. SyMRI Neuro is an FDA- and CE-approved tool providing brain and myelin segmentation and has been technically and clinically validated in MS populations [47–50]. STAGE (strategically acquired gradient echo) is an FDA-approved quantitative MRI-based reporting tool providing atrophy and MS lesion characterization using susceptibility-weighted images. STAGE has been validated on healthy subjects and several MS cases [51, 52].

#### Included tools

The 10 companies and their QReports identified using the search strategy described in the “Methods” section and illustrated in Fig. 1 are presented in Table 1 along with key technical details.

**Table 1:** A database of the QReports and their key technical details presented in alphabetical order of vendor name

Vendor	Product name	CE/FDA status	Approval received	Target disorder(s)	Input sequences	Brain/lesion segmentation/volumetry method	Lesion filling	Brain atrophy data	Cross sectional + longitudinal brain volumetry analysis	Normative reference database	Segmentation Overlays	Methods to account for inter-scanner variability	Image quality control	Deployment procedure
AIRAmed www.airamed.de	AIRAscore	CE – class I	Aug-2020	All neurodegenerative diseases with brain volume reduction, e.g., dementia and MS	3D T1, 3D T2-FLAIR	In-house and CNN-based	Not required	ml, % TIV and normative percentile	Both, indirect longitudinal comparison	8000 + healthy subjects from private and public datasets, mix of field strength and scanner vendors	Brain and lesion segmentation overlays	Mix of field strength and scanner vendors in the normative reference data. For longitudinal comparisons, it is recommended to only use images from the same scanner sequence combination	Automatic checks for technical image parameters in the DICOM header, image QC for movement artifacts should be performed by the reading physician	Cloud-based PACS integration
Combinostics www.cneuro.com	cNeuro cMRI	CE – class IIa FDA – 510(k) cleared, class II	Sep-2016	Dementia, MS and other diseases where analysis of atrophy or WMHs are of interest	3D T1 and 2D or 3D T2-FLAIR	In-house	Did not disclose	ml, normative percentile for cross-sectional data + annualized atrophy rate and its percentile for longitudinal data from T1	Both, direct longitudinal comparison	~ 2000 subjects from private and public US/Europe datasets, 18–94 y, mix of field strength and scanner vendors	Brain and lesion segmentation overlays	Mix of field strength and scanner vendors in the normative reference data	Automated QC for CNR, abnormal signal intensities and acquisition parameter checks	Cloud-based PACS integration
CorTechs.ai www.cortechs.ai	NeuroQuant MS (also known as LesionQuant)	CE – class IIa, FDA – 510(k) cleared, class II	Aug-2006	Multiple sclerosis	3D T1 and 2D or 3D T2-FLAIR	In-house	Using neighborhood information	ml, % change, % TIV and normative percentile	Both, direct longitudinal comparison	~ 5000 subjects from private and public datasets—age range 3–100 y, equal male/female ratio, acquired using Siemens, GE, and Philips MRI scanners with both 1.5 T and 3 T field strength	Lesion and brain segmentation overlays	Scanner-specific 3D gradient field distortion correction; voxel intensity normalization; custom dynamic atlas–based contrast adjustment by anatomical region to correct the measured contrast variability in patients	Automated QC checks for acquisition parameter, atlas fit, atlas tissue class contrast, scan noise estimation and image quality	PACS integration via local hardware, local virtualization or cloud based
Icometrix www.icometrix.com	icobrain ms (previously MSmetrix)	CE – class I, FDA – 510(k) cleared, class II	Jul-2015	Multiple sclerosis	3D T1 and 2D or 3D T2-FLAIR	In-house	Gaussian smoothed mean WM values	ml, % change, normative percentile	Both, direct longitudinal comparison	1903 healthy subjects (1069 female and 834 male subjects) available from several public collections, 6–96 y, mix of field strength and scanner vendors	Lesion and brain segmentation overlays	Cross-sectional report has been tested across multiple scanners. Longitudinal comparisons require same scanner and acquisition protocol for accuracy	Automated flagging for manual QC: incomplete head coverage, insufficient CNR or distortions between sequences or time points	Cloud-based PACS integration, optional EMR integration
jung diagnostics GmbH www.jung-diagnostics.de	Biometrica	CE – class I	Jun-2009	Multiple sclerosis, dementia, neurodegenerative diseases	2D/3D MPRAGE and 2D/3D T2-FLAIR and 2D/3D Gd enhancing T1	In-house, CNN-based and SIENA for longitudinal comparison	Using neighborhood information	z-score using residuals method	Both, direct longitudinal comparison	~ 2000 subjects from a proprietary dataset using a single scanner and protocol, 18–99 y	Lesion and brain segmentation overlays	Site qualification process—protocol set-up using 30 healthy scans for each individual scanner using the service. Manual checks for global offset between each site and proprietary normative database	Site qualification process and expert manual QC/QA checks by vendor	Cloud-based PACS integration
mediaire www.mediaire.de	mdbrain	CE – class I	Jan-2019	Multiple sclerosis, dementia, NPH, neurodegenerative diseases, vascular dementia, brain aneurysm, brain tumor	2D or 3D T1 and T2-FLAIR	In-house	Not required	ml and normative percentile (in %)	Both, direct longitudinal comparison	~ 8000 scans from mainly private datasets, 18–93 y, mix of field strength and scanner vendors	Lesion and brain segmentation overlays	Mixture of field strength and scanner vendors in the segmentation algorithm training data	Automated checks for acquisition parameters and artifact	PACS integrated via local hardware or local virtualization
Pixyl www.pixyl.ai	Pixyl.Neuro.MS Pixyl.Neuro.BV	CE – class IIa	Nov-2019	Multiple sclerosis, neurodegenerative diseases, dementias, Alzheimer’s disease, Parkinson’s disease	Pixyl.Neuro.MS: 2D or 3D T2 FLAIR; 3D T1 and 3D T1 with contrast optional Pixyl.Neuro.BV: 3D T1	In-house, deep learning-based	T1 white matter hypo-intensity segmentation is incorporated as white matter labels	Pixyl.Neuro.BV: ml and in %	Both, indirect longitudinal comparison	Pixyl.Neuro.BV: ~ 3000 subjects from private and public datasets, 18–97 y, multi-center and multi-country (mix of field strength and scanner vendors)	Lesions labeled according to their individual evolution	Data augmentation using proprietary 3DT1 library with clinically relevant MRI and patient variability (noise, contrast, artifacts, distortions, style transfer)	Automated QC based on voxel size, DICOM headers, and acquisition parameters. The user is warned when insufficient input image quality could impact quality of results	PACS integration via local hardware or local virtualization. Stand-alone web based; Integration via AI marketplace and distribution platforms
Quibim www.quibim.com	Quibim Precision: (white matter lesions; atrophy screening)	CE – class IIa	Dec-2018	Neurodegenerative diseases, multiple sclerosis, dementia Parkinson’s disease, epilepsy, amyotrophic lateral sclerosis (ALS)	White matter lesions: 2D or 3D FLAIR; atrophy screening: 3D T1	In-house, lesions: CNN-based; atrophy: atlas-based	Not done or not applied	Atrophy screening: ml, %TIV, normative percentile	Both, indirect longitudinal comparison	620 Caucasians from private and public datasets, 20–86 y, mix of field strength and scanner vendors	Lesion and brain segmentation overlays	Onboarding with sample of new site data, amendments to data pre-processing/acquisition protocols where necessary	Automated QC checks on required acquisition parameter ranges	PACS integrated via local hardware, local virtualization or cloud-based
Qubiotech www.qubiotech.com	Neurocloud VOL	CE – class I	Jan-2019	Multiple sclerosis, epilepsy, dementia, Alzheimer's disease, Parkinson's disease	Cross-sectional: 3D T1 recommended (3D T2-FLAIR also possible) Longitudinal: multi-sequence only (3D T1 and T2 FLAIR) analyses	In-house and SPM-based	Using neighborhood information	ml, %TIV and normative percentile	Both, direct longitudinal comparison	~ 852 subjects from private dataset, age 19–94 y (375 male, 477 female), mix of scanner vendors and field strength	Lesion and brain segmentation overlays	Mixture of field strength and scanner vendors in the normative reference, training, and validation data	Automated QC checks on required acquisition parameters, DICOM headers, and cross-correlation metrics to evaluate image co-registration results	Locally virtualized or cloud-based PACS integration
Qynapse www.qynapse.com	QyScore	CE – class IIa, FDA – 510(k) cleared, class II	CE 2017 FDA 2019	All central nervous system diseases	3D T1 and 2D or 3D T2 FLAIR	Lesion: in-house; atrophy: open source	Not done or not applied	ml, %ICV, z-score, normative percentile	Both, indirect longitudinal comparison	20–90 y, mix of field strength and scanner vendors	Lesion and brain segmentation overlays	Specific MRI parameters are required which have been tested to provide good QyScore results. Early manual QC checks by vendor for each site	Automated QC checks on required acquisition parameters, SNR, CNR	PACS integrated via local hardware or cloud-based

Information gathered from the literature and direct contact with vendors can be assessed according to individual needs. All information was checked and confirmed with vendors before publication. It was not possible for the authors to independently verify technical details without access to commercial software packages

*MS*, multiple sclerosis; *CE*, Conformitè Europëenne; *FDA*, Food and Drug Administration; *FLAIR*, fluid-attenuated inversion recovery; *SIENA*, Structural Image Evaluation, using Normalisation, of Atrophy; *SPM*, statistical parametric mapping; *CNN*, convolutional neural network; *WMH*, white matter hyperintensity; *SNR*, signal-to-noise ratio; *CNR*, contrast-to-noise ratio; *QC*, quality control; *PACS*, picture archiving and communication system; *TIV*, intracranial volume; *CSF*, cerebrospinal fluid; *GM*, grey matter; *WM*, white matter; *DICOM*, Digital Imaging and Communications in Medicine; *QA*, quality assurance

### Company and product features

Table 1 is a structured database of the technical features and characteristics of the QReports. Company and product features are summarized below. Report processing times were not included, as measurement and comparison should be conducted independently by the authors using the same cases and resources, which was not possible without access to the software packages.

#### CE/FDA approval status

All 10 companies have obtained either CE class I/II or FDA 510(k) clearance, as “software as a medical device.”

#### Date of approval

CorTechs.ai was the first company to receive FDA clearance in 2006. The most recent of the 10 companies to receive CE or FDA regulatory approval was AIRAmed in 2020.

#### Target disorder

All companies produced a report for the assessment of MS lesions and brain atrophy. For some tools, the MS QReport was an extension of a previously established brain volume quantification tool.

#### Input sequences

Most companies required 3D T1 and 2D or 3D T2-FLAIR input sequences for brain and lesion segmentation. Two tools also provided the option to use a 2D or 3D gadolinium-enhancing T1-weighted sequence for contrast-enhancing T1 lesion detection.

#### Brain/lesion segmentation/volumetry method

All companies used proprietary methods developed “in house,” of which four claimed to use deep learning. Two companies reported using modified versions of research methods, including SIENA(X)[40] and SPM (www.fil.ion.ucl.ac.uk/spm). Brain and lesion segmentation software was commercialized as a single package or as two different tools (which is the case for two companies, see Table 1).

#### Lesion data

All tools reported longitudinal lesion volume. Nine out of ten tools reported longitudinal lesion count. Nine out of ten QReports provided the spatial distribution of lesions according to the McDonald criteria [53] categorized into periventricular, juxtacortical, deep white matter, and infratentorial. Companies that have not yet included lesion count and spatial distribution of lesions claimed to be working to provide this information in upcoming updates and releases of the tools.

#### Lesion filling

Lesion filling is commonly used to accurately compute brain volumes in MS [54]. Eight out of ten companies used either automatic lesion filling or deep learning approaches, which did not require lesion filling. The approaches used for lesion filling are outlined in Table 1.

#### Brain atrophy data

Brain atrophy was reported in milliliters, as a percentage of the total intracranial volume (TIV), as a normative percentile, or as a z-score.

#### Cross-sectional and longitudinal brain volumetry analysis

All 10 companies provided both cross-sectional and longitudinal analyses of lesions and whole brain atrophy. Longitudinal analysis approaches were indirect for four QReports, i.e., the difference in volume/percentile per structure between two visits and direct for six QReports, such as using SIENA [40].

#### Details of a normative reference population

The normative reference populations of all tools comprised a large age range, typically from 20 to 90 years with a sex balance, and were compiled from public and/or private datasets. Nine out of ten companies used datasets with a range of scanner types and field strength. The size of the datasets varied between 620 and ~8000 subjects.

#### Segmentation/atrophy visual overlays

All QReports provided visual lesion and brain segmentation overlays.

#### Image quality control method

All tools used image quality control (QC) processes. The methods used varied and were mainly automatic, including checks for artifacts and acquisition parameters, computing of standard measures of image quality, such as signal-to- noise ratio (SNR) (comparing the level of the target signal to background noise), and automatic flagging of the need for manual QC.

#### Strategies to account for inter-scanner variability

All companies claimed to use strategies to account for diverse input data, including a mix of scanner type and field strength in the normative reference population, algorithm training, using independent validation datasets, accounting for vendor-specific acquisition parameters, implementing AI-based augmentation to anticipate the variability between images, and using site qualification procedures.

#### PACS integration/report deployment procedure

All companies claimed to provide PACS integration either using a cloud-based solution and/or local virtualization and/or local hardware.

#### Peer-reviewed technical and clinical validation

All companies had conducted internal validation processes, including the necessary steps for CE and/or FDA clearance. Also, all companies claimed to be carrying out further peer-reviewed validation studies. It is of note that several companies had conducted studies validating their tools in other disease areas [55–64]. These papers have only been included if lesion, or brain and lesion, quantification techniques were under investigation in MS and if the tool is commercialized for use in MS either on the company website or in publications.

**Fig. 2:**
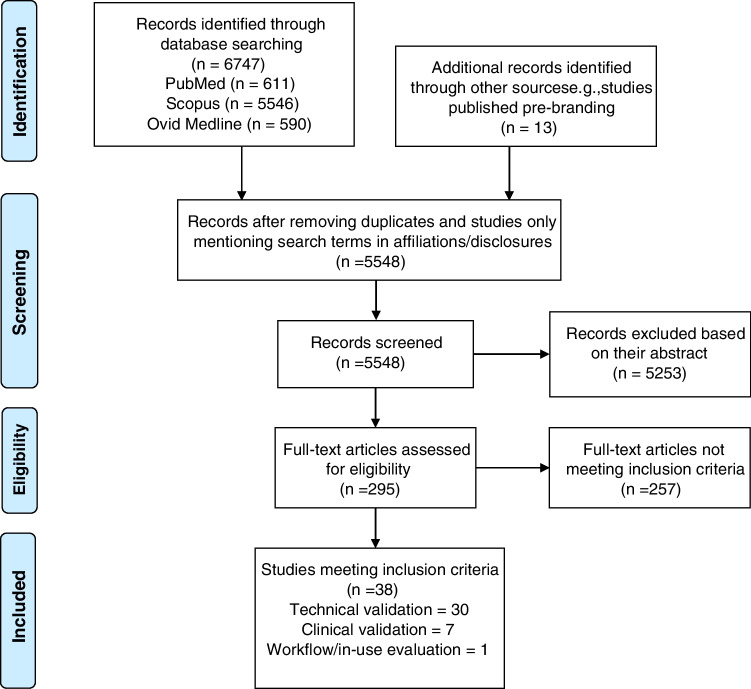
PRISMA flow diagram outlining the search and selection for publications included in the review

The number and category of studies identified in the literature search are presented in Fig. 2 and described below in the “Literature search” section.

### Literature search

**Fig. 3:**
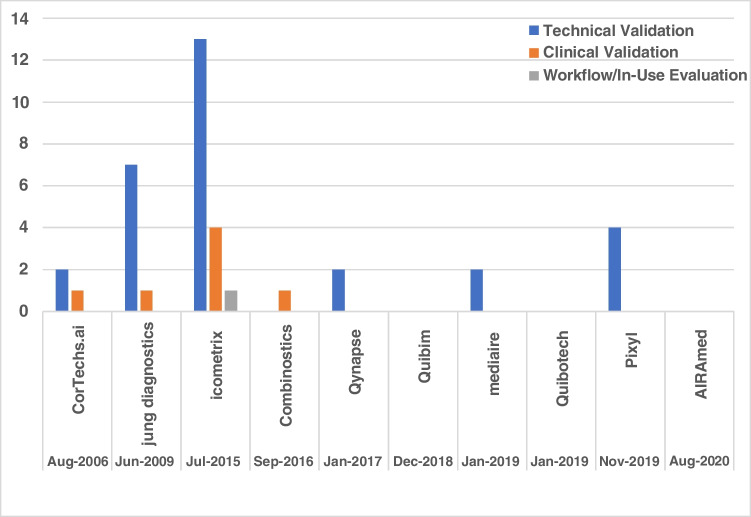
The distribution of publications included in the review for each company identified. The vendors are listed chronologically according to the date of their first FDA/CE approval

The results of the literature search are outlined in the PRISMA workflow diagram in Fig. 2 and documented further below. A total of 38 peer-reviewed publications covering technical (*n* = 30), clinical (*n* = 7) validation, or in-use evaluation (*n* = 1) were identified. In total, 6 companies have conducted technical validation, 4 have published clinical validation, 1 has conducted an in-use evaluation, and 3 have not published studies meeting our inclusion criteria. The distribution of studies identified is presented in Fig. 3.

### Validation studies identified

To remain unbiased, a narrative synthesis of the studies identified for each company is provided and referenced below (in alphabetical order). All publications were conducted in MS and/or healthy control populations. In summary, technical validation mainly consisted of comparison with manual lesion counting, manual segmentation, or state-of-the-art automated brain volumetry and lesion segmentation tools, including SIENA(X) [40], Freesurfer [39], SPM (www.fil.ion.ucl.ac.uk/spm), FIRST [65], Cascade [66], LST [41, 42], Lesion-TOADS [67], lesionbrain [68], BIANCA [69], and nicMSlesions [43]. Technical validation was also conducted as repeatability studies and by testing different acquisition protocols. Clinical validation mainly comprised correlation of the tool’s results with clinical variables, such as EDSS scores. Only one clinical validation study incorporates clinician end-user testing [18]. Validation studies conducted by each company are summarized below.

#### Combinostics

*Clinical*: The cNeuro cMRI results were correlated with EDSS scores [70].

#### CorTechs Labs

*Technical*: NeuroQuant MS has been tested for longitudinal consistency [20] and compared to visual radiological assessment [20], icobrain ms [28], and established brain and lesion segmentation methods (FIRST [71], LST [71], SIENA(X) [28], FreeSurfer [20], and Cascade [20]).

*Clinical*: NeuroQuant MS results were correlated with clinical variables, including EDSS and timed 25-foot walk test (T25FT) and 9-Hole Peg Test (9HPT) scores as measures of MS-related physical disability [20].

#### Icometrix

*Technical*: icobrain ms has been tested for robustness to different input data [26, 31, 33, 72–74], reproducibility [26, 31, 72, 74], repeatability [73, 74], and consistency over time [19, 73]. The tool has been compared to manual segmentation [19, 21, 26, 31, 74, 75], LesionQuant [28], and automated established brain and lesion segmentation and atrophy quantification methods, such as SIENA(X) [28, 72–74, 76, 77], LST [26, 31], Lesion-TOADS [26], FreeSurfer [77], and SPM [74, 77] and has been included in a longitudinal MS lesion segmentation challenge [21]. Earlier or other versions of the tool have been compared to the current version [31, 75]. An automated method to decrease the effect of inter-scanner variability on results has been tested [78, 79].

*Clinical*: The tool has been tested by clinicians as end-users investigating the impact on intra- and inter-rater variability, reporting time (outside of their normal clinical routine), and detection of disease activity in comparison to visual radiological assessment [18]. The ability of the tool to differentiate MS clinical phenotypes has been investigated [18]. The tool’s results were correlated with EDSS [18, 76, 80, 81] and SDMT scores [81] and the number of relapses [80].

*In-use evaluation*: Icometrix has investigated the health-economic impact of icobrain ms in a microsimulation study with a decision analytical model based on a hypothetical cohort of MS patients testing for disease detection, treatment decision-making, patient quality of life, and costs using the tool in comparison to clinical and visual radiological assessment [32].

#### Jung diagnostics

*Technical*: Jung diagnostics have compared the current method to earlier versions of the tool (which included LST) [22, 27, 82, 83] and to manual segmentation [22, 27, 82]. The tool has been tested for repeatability [27, 84, 85], reproducibility [22, 27], and robustness to different input data [22, 27, 86]. Jung diagnostics has compared two methods for brain atrophy data adjustment for head size and age [86].

*Clinical*: Biometrica results were correlated with clinical variables, including EDSS and SDMT scores, disease duration, and MS phenotypes [87].

#### Mediaire

*Technical*: The tool has been tested for robustness to different input data [88] and was compared to manual segmentation and other lesion segmentation tools in a longitudinal MS lesion segmentation challenge [23].

#### Pixyl

Pixyl.Neuro.MS is an MS lesion segmentation tool and Pixyl.Neuro.BV can be used for brain volumetry. *Technical*: The lesion segmentation method has been compared to manual segmentation [24, 29, 89] and to older established automated methods [24, 29, 89], including in an MS lesion segmentation challenge [24]. Newer improved versions of the tool have been compared to previous versions [90] and to manual segmentation [90].

#### Qynapse

*Technical*: Qynapse has compared the current method for lesion segmentation to a previous method, to state-of-the-art lesion segmentation methods (including LST, Lesion-TOADS, lesionBrain, BIANCA, and nicMSlesions), and to manual segmentation [25, 30]. QyScore has been tested for robustness to different input data [30].

## Discussion

This systematic review identified 10 companies currently offering FDA- and CE-cleared QReports for use in MS. Most tools identified in this review have obtained regulatory approval in the last 5 years. By reviewing commercial QReports in MS and previously in dementia [35], we aimed to provide the information needed by clinicians to navigate the rapidly developing market for quantitative reporting tools. Studies identified in this review have been categorized according to the QNI model framework to encourage the adoption of a common translational pathway with rigorous and structured testing. We have identified 38 relevant validation and evaluation studies: 30 technical validation studies, 7 clinical validation studies, and 1 in-use evaluation. In total, 6 QReports have evidence of technical validation, 4 companies have conducted clinical validation, and 1 has conducted in-use evaluation. The date of approval of tools did not always correlate with the number of validation studies identified. For example, CorTechs.ai, which received FDA approval in 2006, began developing and validating their tools in MS after validation in other diseases, such as dementia. Clinical validation studies were more prevalent for companies that had received regulatory approval earlier. All companies claimed to be conducting (further) validation studies.

Previous reviews of MS QReports compare both the methodologies used in research and commercially available tools without naming vendors—mainly due to publication prior to their branding [11, 17, 91–95]. In this paper, we review all identified commercial MS QReports that offer a combination of lesion and brain segmentation and volumetry. We aimed to remain unbiased by synthesizing and categorizing papers avoiding direct comparison and evaluation. There is little scope and evidence to recommend one commercial MS QReport over another, as the needs of purchasers may vary and tools have mainly not been tested under the same conditions using the same database (other than in one study identified in this review, which directly compares the performance of two commercial MS QReports) [28].

Our review has highlighted a lack of clinical validation of MS QReports and in particular testing of tools by clinicians. Only four out of ten vendors had conducted clinical validation in an MS population and three of these companies correlated QReport results with clinical variables without directly involving clinicians in the use of the tool. Correlation with clinical variables, such as EDSS, is a first clinical exploration only and can be successful without demonstrating clinical utility. Only one company has tested the tool by clinician end-users investigating reporting time (outside of normal clinical routine), diagnostic accuracy, and intra- and inter-observer variability [18]. We have demonstrated that testing by clinicians in a clinical context is extremely scarce. In 2021, Pemberton et al also demonstrated a lack of clinical validation of dementia QReports [35]. Clinical validation is part of step 4 of the QNI model framework, which encourages studying the impact of QReports on intra- and inter-rater reliability, diagnostic confidence and accuracy, and clinical management, such as reporting time within the context of normal clinical routine, to promote user-confidence and evidence-based care [34]. The collaboration of clinicians and vendors is key for refining these tools, increasing their clinical uptake, and aiding future developments.

This review has demonstrated a lack of in-use evaluation, which is set out as step 6 of the QNI framework [34]. Only one in-use evaluation study was identified, which is a microsimulation investigating the health economic impact of a QReport in a hypothetical cohort of MS patients [32]. Socioeconomic validation may encourage clinical translation, as the added value for stakeholders such as insurers should be demonstrated to encourage reimbursement for widespread clinical use. The effect of an MS QReport on treatment choice and escalation was explored in the simulated in-use evaluation study; however, this should be further explored in a real-life clinical context [32]. The patient perspective on their digital MS care pathway has been investigated by the same company [18]. Patient-reported outcome measures (PROMs) could be incorporated into in-use evaluation, especially if patients have access to results. Several companies claimed that patient access can be provided in the form of a simplified patient-oriented report and icometrix has developed the icompanion patient app, which provides access to their MRI scans. It is of note that seven out of ten companies have received regulatory approval in the last 5 years and clinical use of commercial QReports is still limited; therefore, in-use evaluation may become more prevalent over time. Presenting the evidence as we have done in this review is important for informed implementation in clinical settings, which in turn may facilitate an increase in opportunities for in-use evaluation.

Conducting clinical validation and in-use evaluation could help companies optimize their tools for application in different clinical settings using diverse input data. Step 5 of the QNI framework focuses on workflow integration, including overcoming barriers to generalizability [34]. All companies provided some form of PACS integration and DICOM standard data format. QReports should be tested for robustness to different scanners and field strengths and the normative reference data provided for contextualization of results should be generalizable, as input data can vary. Vendors had mostly compiled large datasets of normative reference values; however, only one company had compared their control population dataset intercontinentally [33]. The tools identified typically rely on 3D MRI input sequences, which are becoming increasingly available (as recommended by imaging guidelines [96, 97]) but may not yet be used in many clinical settings. Furthermore, there is a discrepancy between the MRI sequences used in standard clinical routine and in commercial QReports for use in MS, as most identified QReports rely on both T1-weighted and T2-FLAIR sequences; however, non-contrast T1-weighted images are not routinely included in the imaging guidelines for MS [5]. Companies should continue to be transparent about the generalizability of their tools and clinical usability should be studied to address translational barriers. Table 1, the database of technical features and characteristics, demonstrates the variation in generalizability measures and can help clinicians select the most appropriate tool for a specific clinical setting.

Structured validation and evaluation procedures could facilitate comparison between tools and their improvement. The QNI framework can provide a structure and guidelines for future studies, especially by highlighting the need for the testing of tools by clinicians [34]. In January 2021, the FDA published a regulatory framework action plan for artificial intelligence/machine learning as a medical device [98] and in October 2021, the FDA, Health Canada, and the UK’s Healthcare products Regulatory Agency (MHRA) defined 10 guidelines for Good Machine Learning Practices (GMLP) [99], which reference testing in a clinical setting and validation of robustness and generalizability. The EU has recently introduced new clinical evaluation requirements for regulatory-approved medical devices [100, 101] and in April 2021, the European Commission published the Artificial Intelligence Act to stimulate the development of AI and ensure its trustworthiness focusing on investment and policy [102]. Furthermore, in March 2022, Icometrix received the first Medtech Innovation Briefing in MS by NICE, which provides advice on use and a summary of the evidence (https://www.nice.org.uk/advice/mib291/chapter/summary). By addressing the testing of QReports and providing guidance for use, regulatory bodies could support transparency and encourage structured validation and evaluation procedures.

### Limitations

Different search strategies were required to identify FDA- and CE-cleared tools. Without a fully searchable database of CE-marked tools, it is possible that tools could have been missed. It is possible that a tool may have been granted FDA or CE regulatory approval or a company published relevant studies during the publication process of this review. The conclusion remains unchanged that there is a lack of clinical validation and in-use evaluation of MS QReports. Some technical information on tools was provided by the companies and could not be independently verified by the authors without access to the software packages.

## Conclusion

This review has identified 10 commercially available MS QReports. We have summarized validation and evaluation studies and provided a database of technical details of the tools to increase transparency and aid evidence-based decision-making in the clinic. We used the QNI framework to classify validation and evaluation studies to promote a common, structured pathway for clinical translation. We revealed an evidence gap in the clinical validation and in-

use evaluation of QReports for use in MS, especially in studies testing the use of the tool by clinicians. In total, 10 companies producing commercial MS QReports were identified, of which 4 have conducted clinical validation (only one study involving clinician end-user testing), and 1 in-use evaluation. With this review, we aim to encourage rigorous, structured testing of QReports to elucidate how these tools can be integrated into clinical workflow for the assessment of MS.

## Data Availability

This review paper covers public data and data provided by the companies.

## Abbreviations

MRI: Magnetic resonance imaging
MS: Multiple sclerosis
QReport: Quantitative volumetric reporting tool
FLAIR: Fluid-attenuated inversion recovery
QNI: Quantitative Neuroradiology Initiative
PRISMA: Preferred Reporting Items for Systematic Reviews and Meta-Analyses
PROSPERO: Prospective Register of Systematic Reviews
FDA: Food and Drug Administration
CE: Conformité Européenne (French for “European conformity”)
EDSS: Expanded Disability Status Scale
SDMT: Symbol Digit Modalities Test
WMH: White matter hyperintensity
SPM: Statistical parametric mapping
LST: Lesion Segmentation Tool
samseg: Sequence Adaptive Multimodal SEGmentation
SIENA(X): Structural Image Evaluation, using Normalisation, of Atrophy
BaMoS: Bayesian Model Selection
Lesion-TOADS: Lesion TOpology-preserving Anatomical Segmentation
BIANCA: Brain Intensity AbNormality Classification Algorithm
T25FT: Timed 25-foot walk test
9HPT: 9-Hole Peg Test
NICE: National Institute for Health and Care Excellence

## Code availability

The script for keyword searching in the FDA database is available at https://github.com/hughp1/FDA_KeyWordSearch/tree/main.
